# Prostate puzzle: Unconventional prostatic STUMP findings in a middle-aged patient prompt closer examination

**DOI:** 10.1016/j.radcr.2024.10.104

**Published:** 2024-11-22

**Authors:** Mohammadrzea Elhaie, Abolfazl Koozari, Mohammad Reza Khademi

**Affiliations:** a Department of Medical Physics, School of Medicine, Isfahan University of Medical Sciences, Iran; b Department of Medical Physics, School of Medicine, Ahvaz Jundishapur University of Medical Sciences, Iran; c Department of Radiooncology, School of Medicine, Cancer Prevention Research Center, Seyyed Al-Shohada Hospital, Isfahan University of Medical Sciences, Iran

**Keywords:** Prostatic neoplasms, Neoplasms, Magnetic resonance imaging, STUMP

## Abstract

A 41-year-old male presented with obstructive urinary symptoms and an enlarged prostate. Subsequent testing revealed a remarkably high PSA level of 150 ng/mL, considerably above normal limits, raising concern for possible malignancy. Transrectal ultrasound showed an enlarged heterogeneous prostate measuring 74×80×75mm. PSMA PET/CT detected intense tracer uptake. While MRI and biopsy ruled out carcinoma, they indicated Stromal Tumors of Uncertain Malignant Potential (STUMP), an uncommon diagnosis in younger patients. This complex case highlights the essential role of advanced multimodal imaging and immunohistochemistry in diagnosing atypical prostatic lesions of uncertain malignant potential like STUMP, given their histologic similarity to benign conditions posing diagnostic difficulties. A multidisciplinary approach led to the STUMP diagnosis and decision for long-term monitoring instead of radical prostatectomy due to STUMP's unpredictable behavior. This report reinforces the need for diligence when evaluating seemingly benign but diagnostically challenging presentations that could conceal conditions like STUMP, and the importance of a multidisciplinary approach for accurate diagnosis and management of such rare prostatic proliferations.

## Introduction

Stromal Tumors of Uncertain Malignant Potential (STUMP) are infrequent neoplasms, distinguished by an idiosyncratic and atypical proliferation of prostate stroma [1,2]. The pathological evaluation and differentiation of STUMP often present substantial challenges, as this entity shares a histopathological similarity with benign forms of stromal proliferation.

## Case presentation

**Fig. 1:**
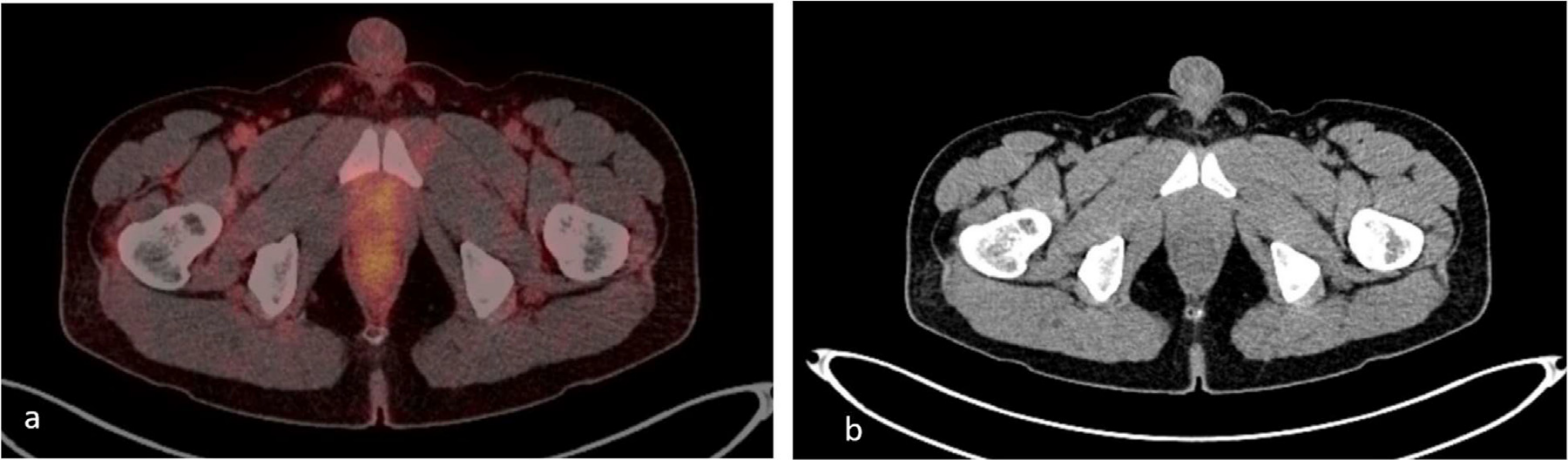
(A) Axial PSMA PET/CT imaging of the prostate and (B) axial CT imaging of the prostate.

A 41-year-old Middle Eastern man with an unremarkable health history was admitted to the urology department, presenting with urinary retention and nocturia. His physical assessment was largely normal, with the exception of noticeable discomfort upon palpation in the lower abdominal region. An initial pelvic sonography unveiled a significantly enlarged prostate, measuring 74×80×75 mm, featuring irregular wall thickening and a heterogeneous echotexture, raising initial concerns for possible pathological conditions of the prostate.Subsequent laboratory analysis revealed a remarkably high PSA level of 150 ng/mL, considerably above the upper limit of the normal range, which further underscored the severity of the patient's condition [3]. Additional blood work reported an elevated erythrocyte sedimentation rate (ESR) of 62 mm/L and a notable vitamin D deficiency, both of which suggested an underlying inflammatory process and raised the suspicion of a potential malignancy [4]. Given the abnormal findings and in pursuit of a definitive diagnosis, a broader diagnostic strategy was employed. This entailed a repeat sonography with a specific focus on the penile region to assess the integrity of the penile vasculature and erectile function. The penile sonography meticulously evaluated the intracavernosal arteries and erectile response, which were found to be entirely within normal parameters, thus ruling out vascular contributions to the patient's urinary symptoms. Despite the lack of conclusive evidence pointing towards a malignant condition from the initial assessments, the patient was subjected to further imaging studies to explore all possible underlying causes of his symptoms. An fluorodeoxy glucose -positron emission tomography (FDG PET) scan was conducted, which yielded no significant findings, leaving the clinical team at a diagnostic crossroads. In a subsequent attempt to pinpoint the cause of the patient's enlarged prostate and elevated PSA levels, a prostate-specific membrane antigen positron emission computed tomography (PSMA PET/CT) scan was performed (Fig. 1). The results were striking, revealing a markedly enlarged prostate gland with intense Ga-PSMA uptake, a finding that was highly suggestive of a neoplastic process. This imaging modality provided a crucial piece of information that contrasted with the benign nature of the patient's clinical presentation and biopsy results, warranting a deeper investigation into the nature of the prostatic enlargement.

**Fig. 2:**
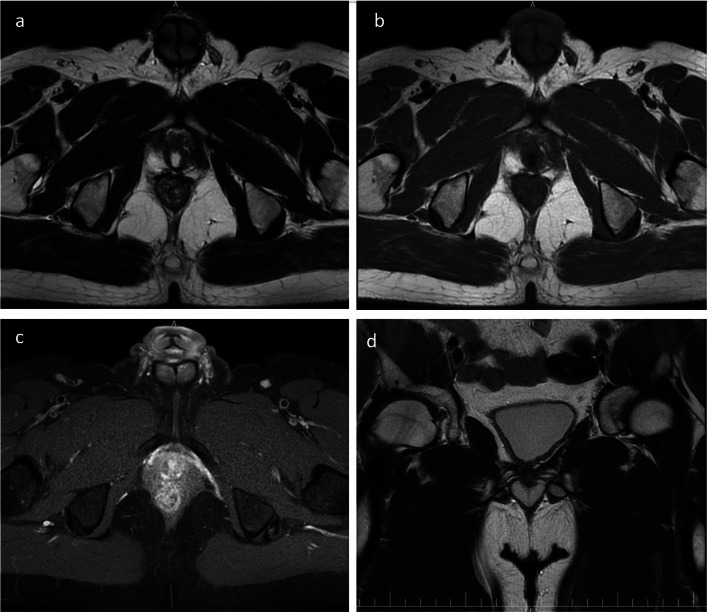
MRI of the prostate: (A) axial T2-weighted, (B) axial T1-weighted FSE, (C) axial T1-weighted with contrast enhancement (CE), and (D) coronal T2-weighted.

Following the PET\CT Imaging, the patient underwent a comprehensive series of multi-sequence Magnetic resonance imaging (MRI) scans of the prostate. This progressive imaging approach was vital to further delineate the extent and nature of the pathology, thereby enriching the diagnostic trajectory with invaluable insights and guiding towards a more targeted pathological examination (Fig. 2). To ascertain the precise nature of the cellular proliferation observed, the patient underwent a series of immunohistochemical (IHC) tests on the biopsy samples retrieved from the prostate gland.

The immunohistochemical analysis employed a panel of antibodies targeting specific markers known to delineate the characteristics of various prostatic lesions and their potential malignancy. This assessment was pivotal, considering the ambiguity surrounding the patient's clinical presentation and the high PSMA uptake indicating a possible malignant entity. IHC tests are renowned for their ability to provide a vivid and detailed cellular landscape, thus facilitating a more definitive diagnosis by highlighting the expression patterns of certain proteins that are indicative of various types of tissue origin and potential for malignancy [5,6].

## Discussion

The outcome of the immunohistochemical examination was revealing. The tests vividly identified the presence of a STUMP. STUMP lesions are marked by their stromal cell proliferation that defies the conventional benign-malignant classification, hence the designation of "uncertain malignant potential [7]." These tumors are exceedingly rare, which added an additional layer of complexity to the patient's already atypical presentation. Remarkably, the diagnosis of STUMP in a relatively young patient, such as the 41-year-old man in this case, is an even rarer occurrence, as these tumors typically present in an older demographic. The rarity of the condition, coupled with the patient's age, underscored the unique nature of this case, highlighting the indispensable role of immunohistochemical testing in unraveling the intricacies of unusual prostatic proliferations. Multidisciplinary approach and diagnostic workup the complexity of this case necessitated a comprehensive, multidisciplinary approach to ensure an accurate diagnosis and optimal management. The treatment team was composed of a radiation oncologist, pathologist, and urologist, who collaborated closely throughout the diagnostic and decision-making process. Given the concerning findings on the PSMA PET/CT scan, a targeted prostate biopsy was performed to obtain tissue samples for histopathological examination. Contrary to the expected malignant findings, the biopsy results demonstrated characteristics that were inconsistent with prostate carcinoma, instead pointing towards a benign etiology. After a thorough review of all the diagnostic materials, including the imaging studies, pathology reports, and clinical findings, the multidisciplinary team reached a consensus on the revised diagnosis. They agreed to withhold the radiation therapy and instead closely monitor the patient's condition through regular follow-up, utilizing various imaging and pathological tests to detect any potential recurrence or progression of the disease. This multidisciplinary approach, with close collaboration between the specialists, was crucial in arriving at the correct diagnosis and formulating the appropriate management plan for this rare and complex case.

## Conclusion

The diagnosis and management of the case presented herein underscore the intrinsic complexities and diagnostic challenges associated with Prostatic STUMP. This rare pathological entity demands a high degree of suspicion and a thorough diagnostic approach, integrating both advanced imaging techniques and comprehensive histopathological evaluation through immunohistochemical analysis. The patient, a relatively young 41-year-old male, exhibited symptoms and preliminary findings that could easily be misconstrued as indicative of more common prostatic conditions such as Benign Prostatic Hyperplasia (BPH). The initial presentation, alongside imaging studies showing significant prostate enlargement and elevated PSA levels, mirrors the clinical picture often associated with BPH, thereby highlighting the potential for misdiagnosis.

The distinguishing diagnosis of STUMP in this case was challenging yet crucial, as the therapeutic strategies and prognostic outcomes differ markedly from those associated with BPH and other prostatic diseases. The decision to proceed with radical prostatectomy was predicated on the unique nature of the STUMP diagnosis, aiming to mitigate the risk of potential malignant transformation—a risk that is not typically associated with BPH. Postoperative assessments, including laboratory tests showing normalized PSA levels and follow-up imaging, have been instrumental in confirming the success of the surgical intervention and in ensuring the absence of residual disease. This case vividly illustrates the essential role of diligence and specificity in diagnosing unusual prostate conditions, which, if misdiagnosed as more benign entities such as BPH, could lead to suboptimal management and potentially overlook the need for more aggressive treatment modalities. Furthermore, it emphasizes the importance of a multidisciplinary approach in managing complex urologic cases, from accurate diagnosis through to tailored treatment and meticulous postoperative follow-up. The successful outcome of this case serves as an educational cornerstone, reinforcing the need for heightened awareness and diagnostic precision in the face of clinical presentations that may initially appear typical of more common prostatic diseases.

## Patient consent

The patient provided written informed consent for the publication of this case report and accompanying images. All efforts have been made to protect the patient's privacy and anonymity. The patient understands that the information will be published without their name attached, but that full anonymity cannot be guaranteed. They understand that the text and images published in the article will be freely available on the internet and may be seen by the general public. The patient has been offered the opportunity to read the manuscript and has agreed to its publication.
